# Prediction of Fragmentation Pathway of Natural Products, Antibiotics, and Pesticides by ChemFrag

**DOI:** 10.1002/jms.5129

**Published:** 2025-04-07

**Authors:** Jördis‐Ann Schüler, Annemarie E. Kramell, Antonia Schmidt, Pauline D. Walesch, René Csuk

**Affiliations:** ^1^ Institute of Computer Science Martin Luther University Halle‐Wittenberg Halle (Saale) Germany; ^2^ Department of Organic Chemistry Martin Luther University Halle‐Wittenberg Halle (Saale) Germany

**Keywords:** fragment ion annotation, mass spectrometry, natural products, rule‐based fragmentation, semiempirical quantum mechanics

## Abstract

Because the manual interpretation of ESI‐MS ^n^ fragmentation spectra is time‐consuming and usually requires expert knowledge, automated annotation is often sought. The fragmentation software ChemFrag enables the annotation of MS ^n^ spectra by combining a rule‐based fragmentation and a semiempirical quantum chemical approach. In this study, the rule set was extended by 31 cleavage rules and 12 rearrangement rules and used for the interpretation of ESI(+)‐MS ^n^ spectra of antibiotics, pesticides, and natural products as well as their structural analogs. The fragmentation pathways predicted by ChemFrag for compounds such as 17*β*‐estradiol were confirmed by a comparison with pathways published in other studies. In addition, the annotations were compared with those of the programs MetFrag and CFM‐ID , for example, with regard to the number and intensity of annotated fragment ions. Our experiments show that ChemFrag provides reliable and in some cases chemically more realistic annotations for the fragment ions of the investigated compounds. Thus, ChemFrag is a helpful addition to the established in silico methods for the interpretation of ESI(+)‐MS ^n^ spectra.

## 1 Introduction

Mass spectrometry (MS) with their various configurations is unquestionably a powerful tool for the identification and quantification of organic compounds. Depending on the sample introduction system, ionization technique, and mass analyzer, organic compounds with very different properties can be characterized and various issues addressed. Electrospray ionization (ESI)‐MS in combination with liquid chromatography (LC‐ESI‐MS or LC‐ESI‐MS/MS) is used for numerous applications, for example, in medicine or biochemistry. This method is suitable for the characterization of nonvolatile, thermally unstable, or polar compounds even in complex matrices. In addition to other techniques such as nuclear magnetic resonance (NMR) or infrared (IR) spectroscopy, fragmentation (MS ^2^ or MS ^n^ ) spectra in particular can contribute to structure elucidation. Manual interpretation of fragmentation spectra is time‐consuming and usually requires expert knowledge. Thus, an automated interpretation of the generated data, which enables high‐throughput screening, is helpful for many applications. One approach for the identification of unknown compounds is the comparison with spectral libraries [ 1 ] such as MassBank, currently containing 96.350 MS ^2^ spectra, or the NIST and Wiley databases. However, spectral databases based on ESI‐MS/MS are relatively small. The comparison of spectra is further complicated by the fact that device parameters such as collision energy are often decisive for the number and intensity of fragment ion peaks. Another approach for the annotation of MS/MS spectra is the application of in silico methods [ 2 ] . These include rule‐based fragmentation (e.g., *Mass Frontier* HighChem, Ltd. Bratislava, Slovakia, versions after 5.0 available from Thermo Scientific, Waltham, USA) [ 3 ] , combinatorial fragmentation [ 4, 5 ] , comparison of fragmentation trees [ 6, 7 ] , or machine‐learning based approaches [ 8, 9, 10 ] . Established programs are, for example, MetFrag [ 11], CFM‐ID [ 12], or SIRIUS [ 13]. These methods were originally developed for the identification of metabolites based on the measured spectrum. To identify the metabolite, they determine, among other things, the structures of the fragment ions. The advantage of these programs is their short runtime; however, chemically unplausible fragment ions are occasionally generated. In contrast, quantum chemical‐based methods [ 14 ] , such as QCMS ^2^ [15, 16] or QC‐FPT [ 17], form chemically correct fragment ions. However, in many cases, they require a significantly longer runtime to do so. In addition, the required software is usually not available free of charge. Approaches based on quantum chemistry as well as rule‐based fragmentation are combined in the program ChemFrag [18]. With its shorter runtime than established quantum chemical methods and its chemically more plausible annotation than rule‐based fragmentations, ChemFrag provides the basis for the prediction of fragmentation pathways of different classes of organic substances. Furthermore, the semiempirical PM7 method implemented in the Molecular Orbital PACkage program (MOPAC), which is available to users free of charge in an academic context, is used for the quantum chemical calculations.

In this study, we present the further development of ChemFrag for the interpretation of ESI(+)‐MS ^n^ fragmentation spectra of antibiotics, pesticides, natural products, and their structural analogs such as steroids, flavonoids, sulfonamides, and butanoic acid esters. This includes the implementation of new rules and the verification of predicted fragmentation paths. Some fragmentation pathways are compared with those already published, whereas for other compounds, fragmentation ions are annotated for the first time.

## 2 Materials and Methods

### 2.1 ChemFrag Architecture/Workflow

ChemFrag is a tool for the interpretation of MS ^n^ fragmentation spectra that combines a rule‐based approach with quantum chemical calculations (see [ 18 ] ). For the quantum chemical calculations, the semiempirical PM7 method is used, which is implemented in MOPAC. This method provides heats of formation, which are used to select chemically meaningful fragment ions for subsequent fragmentation steps, as well as bond orders for identifying weak bonds. Using the rule‐based approach, ChemFrag generates energetically stable fragment ions. ChemFrag currently incorporates 44 cleavage rules and 16 rearrangement rules, derived from the literature [ 19, 20, 21, 22, 23, 24, 25, 26, 27 ] or quantum chemical computations.

In the following, we will briefly describe the process of fragmentation simulation using ChemFrag . The first step involves the ionization of the input molecule (positive ion mode: protonated molecule ions [M+H] ^+^ ). Subsequently, stable molecule ions are selected for the initial fragmentation step on the basis of reaction enthalpies calculated by the heat of formations of the products and reactants. Next, fragment ions are generated by applying cleavage and rearrangement rules and by cleaving bonds with a low bond order. The chemical plausibility of the formed fragment ions is evaluated using semiempirical calculations. Chemically meaningful fragment ions are selected for the next fragmentation step. This cycle continues until a specified termination criterion, for example, the fragmentation depth, is met.

If an *m/z* value of a generated (fragment) ion matches a signal in the experimentally determined MS ^n^ spectrum, the (fragment) ion is assigned to this peak. ChemFrag 's output includes the assignment of molecule and fragment ions to the experimentally determined *m/z* values as well as a fragmentation tree. The fragmentation tree allows the reconstruction of reaction pathways leading to the formation of fragment ions. The implemented cleavage and rearrangement rules can be extended dynamically. To simulate various ionization strengths, users can adjust parameters such as reaction enthalpies or fragmentation depth. Moreover, the integration of other semiempirical quantum chemical methods such as the GFN2‐xTB method [ 28 ] into ChemFrag is conceivable. However, this is the subject of future studies.

### 2.2 Implementation of new Fragmentation Rules

**SCHEME 1:**
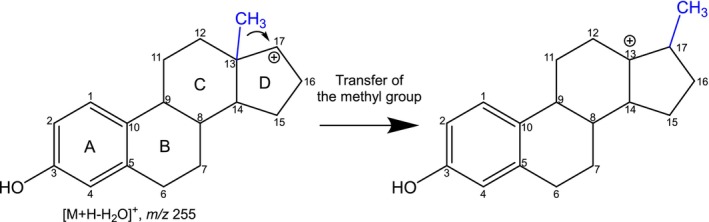
Migration of a methyl residue starting from fragment ion [M+H‐H_2_O]^+^ of **1** ESI(+) rearrangement rule: methyl shift for steroids.

Compared with the first version of ChemFrag , which was used for the interpretation of ESI(+) CID mass spectra of doping substances such as ephedrine and cocaine, the rule set has been expanded [ 18 ] . The cleavage and rearrangement rules implemented in the current ChemFrag version are shown in Tables S1 and S2 as well as in [ 18 ] . The newly implemented rules allow the investigation of further compound classes. One of the newly introduced rules is the methyl shift, which enables a chemically meaningful annotation of MS/MS spectra of protonated steroids ([M+H] ^+^ ). The migration of methyl residues according to a Wagner–Meerwein rearrangement was reported for 10‐, 13‐, and 17‐methyl steroids such as methyltestosterone, methandienone, 5*α*‐androst‐1‐en‐17*β*‐ol‐3‐one, estrone, 17*α*‐estradiol, and 17*β*‐estradiol (**1**) [ 20, 29, 30, 31 ] . As shown in Scheme 1 for **1**, a positive charge at C‐17 triggers the 1,2‐transfer of the methyl group from C‐13 to C‐17, forming a stable tertiary carbocation.

ChemFrag uses a partial structure of the steroid system, consisting of the C‐ and D‐ring, to check the applicability of the methyl shift. In order to keep the rule more general, a six‐membered ring is also permitted instead of the five‐membered ring. Specifically, ChemFrag now checks on the basis of the SMARTS [CH3]C12CCCC1CC[C+]2 and [CH3]C12CCCCC1CC[C+]2 whether a suitable substructure is contained in the molecule. As soon as the substructure test is successful, ChemFrag conducts the following steps for the rearrangement (for the numbering of the C atoms see rearrangement rule 13; Table S2 ):

1. The bond between the C‐1 atom and the methyl group is cleaved.
2. A new single bond is formed between the methyl group and the charged C‐9 atom at the five‐ring or C‐10 at the six‐ring.
3. The charge of the positively charged atom is reduced by one.
4. The charge of the C‐1 atom is increased by one.

### 2.3 ESI‐MS ^n^ Analyses

ESI‐MS ^n^ spectra were recorded using a Finnigan LCQ mass spectrometer (ion source: ESI, cation sensitive detection, spray gas: N_2_ , damping and collision gas: He, CID mass spectrometry). Two acquisition modes were used to characterize each compound: full scan mode and MS ^n^ of the precursor ion. The steroids have been provided by hapila GmbH (Gera, Germany); all other compounds have been a gift by Organica GmbH (Bitterfeld‐Wolfen, Germany).

## 3 Results and Discussion

To evaluate the evolution of ChemFrag , the program was applied to 22 compounds from different substance classes. To verify the effectiveness of the new rules, the experiments were divided into several sections. First the fragmentation pathway of estrogenic steroid **1** as predicted by ChemFrag was compared with a previously published one. Second, the number of annotated fragment ions from ChemFrag was compared with those obtained by established programs such as MetFrag and CFM‐ID . In addition, the newly implemented rules were used to predict the fragmentation behavior of several steroids, such as estriol 3‐methyl ether (**2**) and ∆ ^9,11^ ‐dehydro‐17*α*‐cyanomethylestradiol (**3**). For the latter compounds, the authors are not aware of any ESI(+)‐MS ^n^ fragmentation spectra with annotated fragmentation ions. Consequently, the fragmentation paths of these compounds are predicted for the first time in this study. Finally, other classes of compounds were included in the analysis, and mass spectra of compounds such as 2‐cyano‐2‐phenylbutanoic acid ethyl ester (**4**), nicotinamide (**5**), and the flavonoid quercetin (**6**) were interpreted and annotated by ChemFrag.

### 3.1 Annotation of ESI‐MS ^n^ Spectra of Various Steroids and Comparison to MetFrag and CFM‐ID

**SCHEME 2:**
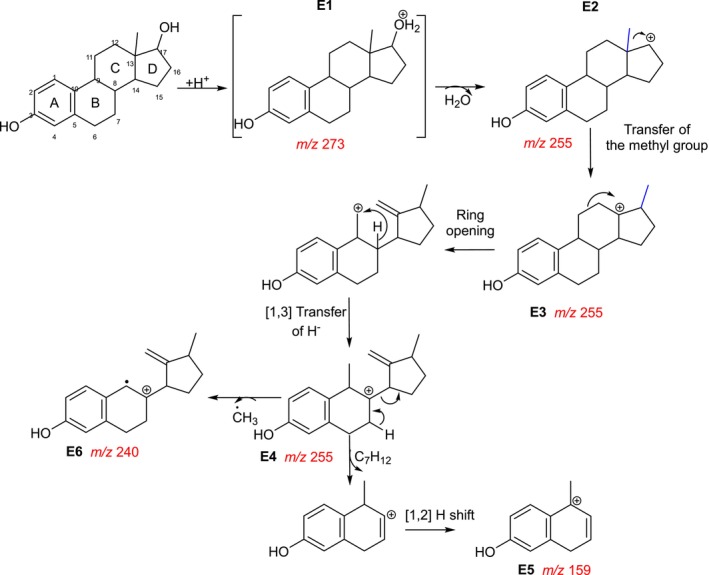
Fragmentation pathway of protonated **1** [M+H]^+^ predicted by ChemFrag [(fragment) ions described by Ma and Yates [20]: *m/z* 273, 255, 159].

In the first experiment, the reaction pathway predicted by ChemFrag for protonated **1** (**E1**, [C_18_ H_25_ O_2_ ] ^+^ , *m/z* 273.19, Scheme 2 ) was compared with that of Ma and Yates of 2018 [ 20 ] , which closely follows the results of Bourcier et al. of 2010 [ 31 ] . In both cases, water elimination is predicted after protonation of the hydroxyl group at C‐17, forming a secondary carbocation (**E2**, [C_18_ H_23_ O] ^+^ , *m/z* 255.17). To generate a more energetically stable tertiary carbocation (**E3**), ChemFrag and Ma and Yates both propose a 1,2‐transfer of the methyl group from C‐13 to C‐17, followed by an opening of the C‐ring. The opening of the C‐ring forms a primary carbocation, which is converted into a tertiary carbocation by hydride transfer (**E4**). This is followed by the cleavage of the D‐ring, a rearrangement of the positive charge to the benzylic position and the formation of the resonantly stabilized naphthalenol derivative **E5** at *m/z* 159.08 ([C_11_ H_11_ O] ^+^ ). In contrast to Ma and Yates, ChemFrag additionally predicts the loss of a methyl radical from the benzylic position of **E4** and the formation of the stable distonic radical cation **E6** at *m/z* 240.15 ([C_17_ H_20_ O] ^+^ ). The loss of an alkyl radical from [M+H] ^+^ ions is known for steroids and has already been described by Guan et al. [ 32 ] . In addition, LC‐ESI(+)‐MS ^2^ spectra of compound **1** available in the MassBank database also show a peak at *m/z* 240.15 [MS analyzer: quadrupole time‐of‐flight (Q‐TOF); collision energy ( ce ): 20–50 eV] [ 33 ] .

In summary, the newly implemented rules for the rearrangement of the methyl group and the opening of the six‐membered ring lead to a reaction pathway that is consistent with the literature. Thus, we have demonstrated that ChemFrag is capable of predicting chemically meaningful annotations and is suitable for the prediction of steroid fragmentation pathways.

Moreover, ChemFrag was tested for other steroids, and the annotation rates of the total of nine steroids were compared with those of MetFrag 2.0 and CFM‐ID 4.0 ( Table 1 , for structures see Figure S1 ). In this experiment, the absolute score and the weighted score were used. The absolute score is the number of annotated peaks out of the total number of peaks in the spectrum (peaks with an rel. intensity ≥ 5% or 10% are taken into account in the evaluation; see Tables 1 and 4 ). The weighted score takes into account the rel. intensity, that is, the annotation of peaks with high intensity has a stronger influence than that of low intensity peaks. The weighted score (*s*) is calculated using the Formula ( 1 ) , which forms the sum over the set of intensities of the annotated peaks (*F*). The total weighted score of a spectrum is the sum over all peak intensities. While the absolute score shows how many peaks of a spectrum we can explain, the weighted score improves mainly when high intensity peaks, not necessarily more peaks, are annotated.

(1) $$s=\sum_{f\in F}{\text{intensity}}_{f}$$

**TABLE 1:** Comparison of the weighted scores and the absolute scores (in brackets) of selected steroids. The annotation of the (fragment) ions was performed with ChemFrag, MetFrag 2.0, and CFM‐ID 4.0 (scores in the format “determined score/maximum score”; peaks with an rel. intensity ≥ 5% are taken into account in the evaluation).

Substance	ChemFrag	MetFrag	CFM‐ID
17*β*‐Estradiol (**1**)	220/220 (4/4)	120/220 (3/4)	210/220 (3/4)
Equilin 3‐acetate	180/188 (4/5)	166/188 (4/5)	14/188 (1/5)
Estriol 3‐methyl ether (**2**)	305/390 (9/16)	34/390 (4/16)	100/390 (1/16)
9(11)‐Dehydroestrone	134/234 (4/5)	140/234 (4/5)	125/234(3/5)
Estriol 3‐acetate	119/227 (4/6)	181/227 (4/6)	38/227 (1/6)
∆ ^9,11^ ‐Dehydro‐17*α*‐cyanomethyl‐estradiol (**3**)	321/343 (5/8)	199/343 (3/8)	100/343 (1/8)
17*α*‐Cyanomethyl‐estradiol	285/325 (5/9)	213/325 (6/9)	100/325 (1/9)
Estriol 17‐acetate	164/164 (4/4)	152/164 (3/4)	12/164 (1/4)
*α* ‐Hydroxyestrone diacetate	205/219 (5/7)	158/219 (5/7)	55/219 (1/7)
Total score	1933/2310(43/64)	1363/2310 (36/64)	754/2310 (13/64)

As shown in Table 1 , ChemFrag annotates the most (fragment) ion peaks with higher intensity. In contrast, the total weighted score of MetFrag for the examined steroids is about 600 lower, which is probably related to the missing annotation of the protonated molecule ion ( [M+H] ^+^ ). In contrast to ChemFrag and CFM‐ID, MetFrag only annotates fragment ion peaks of the respective compound but not the signal of the [ M+H] ^+^ ion. If the signal of the protonated molecule shows a high intensity, this nonexplanation is clearly reflected in the score. In comparison, the total weighted score of ChemFrag is almost three times as high as that of CFM‐ID. The absolute scores of CFM‐ID show that mostly only one or at most three fragment ions of the steroids could be annotated. We therefore conclude from this experiment that the number of fragment ions annotated by ChemFrag is comparable to MetFrag and is higher than CFM‐ID. A comparison of the generated structures also shows that ChemFrag achieves chemically more meaningful results than CFM‐ID. For example, in the case of **1** (C_18_ H_24_ O_2_ ), CFM‐ID generates a fragment ion with a protonated hydroxyl group at C‐3 after the elimination of CH_4_ and H_2_ ([C_17_ H_19_ O_2_ ] ^+^ , *m/z* 255.14, Table 2 ). In comparison, as described above, ChemFrag predicts a loss of water after protonation of the hydroxyl group at C‐17, followed by a 1.2 methyl shift, opening of the C‐ring and hydride transfer, forming the fragment ion **E4** ([C_18_ H_23_ O] ^+^ , *m/z* 255.17; see Scheme 2 ). As described above, the formation of fragment **E4** is consistent with the studies of Ma and Yates [ 20 ] .

**TABLE 2:** Comparison of selected fragment ion structures of **1**, generated by ChemFrag and CFM‐ID .

ChemFrag		CFM‐ID	
*m/z*	Fragment ion	*m/z*	Fragment ion
255.17	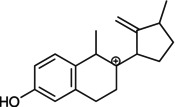 [C_18_ H_23_ O] ^+^	255.14	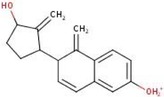 [C_17_ H_19_ O_2_ ] ^+^
159.08	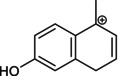 [C_11_ H_11_ O] ^+^	159.08	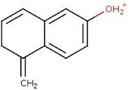 [C_11_ H_11_ O] ^+^

**SCHEME 3:**
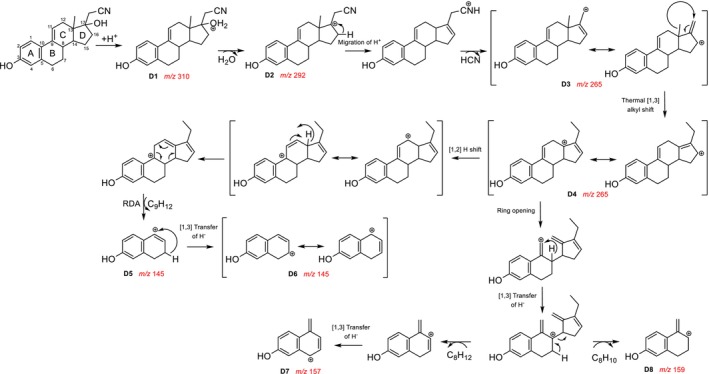
Fragmentation pathway of protonated **3** [M+H]^+^ predicted by ChemFrag.

**FIGURE 1:**
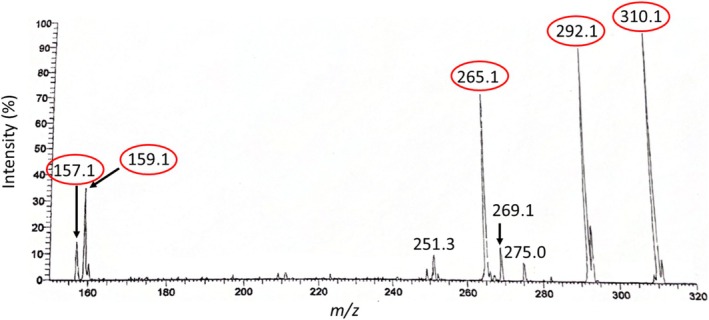
ESI(+)‐MS^2^ spectrum of **3**. The precursor ion [M+H]^+^ at *m/z* 310 and the marked fragment ions were predicted by ChemFrag; *m/z* values used for the calculation of the weighted scores and the absolute scores: *m/z* 310 (100%), 292 (92%), 275 (8%), 269 (12%), 265 (72%), 251 (10%), 159 (35%), 157 (14%).

ChemFrag also achieves very good results for the steroids **2** and **3**. Published fragmentation pathways are not yet available for these substances. Consequently, this is the first time that fragmentation pathways for these structures have been predicted on the basis of ChemFrag (steroid **3**: see Scheme 3 ; steroid **2**: see Scheme S1 ). Fragment ions of these compounds, which were detected in ESI(+)‐MS ^2^ analyses, are given in Table 3 . In the following, a closer look at the fragmentation pathway of the protonated molecular ion of **3** ( Scheme 3 , Figure 1) is performed. Compound **3** is protonated at the hydroxy group in position C‐17 (**D1**, [C_20_ H_24_ NO_2_ ] ^+^ , *m/z* 310.18) followed by water elimination (**D2**, [C_20_ H_22_ NO] ^+^ , *m/z* 292.17). Then, proton transfer and C‐CN cleavage occur, leading to the loss of HCN and the formation of an allylic carbocation at *m/z* 265.16 (**D3**, [C_19_ H_21_ O] ^+^ ). Subsequently, a tertiary carbocation (**D4**) is formed by allylic rearrangement and thermal [1,3] alkyl shift. A subsequent rearrangement of a hydrogen atom to an adjacent carbon atom with concurrent rearrangement of the charge results in an allylic carbocation. The fragment ion **D5** at *m/z* 145.07 ([C_10_ H_9_ O] ^+^ ) is formed by a hydride transfer and a retro‐Diels–Alder (RDA) reaction. A further hydride transfer generates the resonantly stabilized fragment ion **D6**. However, the formation of the fragment ion **D6** cannot be proven experimentally, as data are only available for the *m/z* range 150–320. Starting from the fragment ion **D4**, the fragment ions **D7** at *m/z* 157.06 ([C_11_ H_9_ O] ^+^ ) and **D8** at *m/z* 159.08 ([C_11_ H_11_ O] ^+^ ) can be formed by opening of the C‐ring, formation of a tertiary carbocation by hydride transfer and cleavage of the D ring .

**TABLE 3:** ESI‐MS ^*2*^ data of **2**, **3**, and **4** under ESI(+) conditions (mass range: *m/z* 100–320, 150–320, or 60–220).

Substance	Formula		Fragment ions
		[M + H] ^+^	*m/z*
Estriol 3‐methyl ether (**2**)	C_19_ H_26_ O_3_	303 (100%)	285 (38%), 274 (8%), 267 (70%), 257 (10%), 241 (16%), 227 (13%), 211 (12%), 199 (10%), 185 (26%), 173 (6%), 171 (11%), 151 (12%), 147 (19%), 135 (14%), 121 (25%)
∆ ^9,11^ ‐Dehydro‐17*α*‐cyanomethylestradiol (**3**)	C_20_ H_23_ NO_2_	310 (100%)	292 (92%), 275 (8%), 269 (12%), 265 (72%), 251 (10%), 159 (35%), 157 (14%)
2‐Cyano‐2‐phenylbutanoic acid ethyl ester (**4**)	C_13_ H_15_ NO_2_	218 (47%)	190 (100%), 162 (10%)

### 3.2 Application to Other Substance Classes

In addition to the application to steroids, we are able to successfully confirm published fragmentation pathways for various organic compounds and propose new fragmentation pathways using ChemFrag (for structures, see Figure S3 and Schemes S2 and S3 ). This includes, for instance, the confirmation of the reaction pathway of **5** according to the studies of Hau et al. [ 25 ] and the prediction of a pathway for **4**.

**SCHEME 4:**
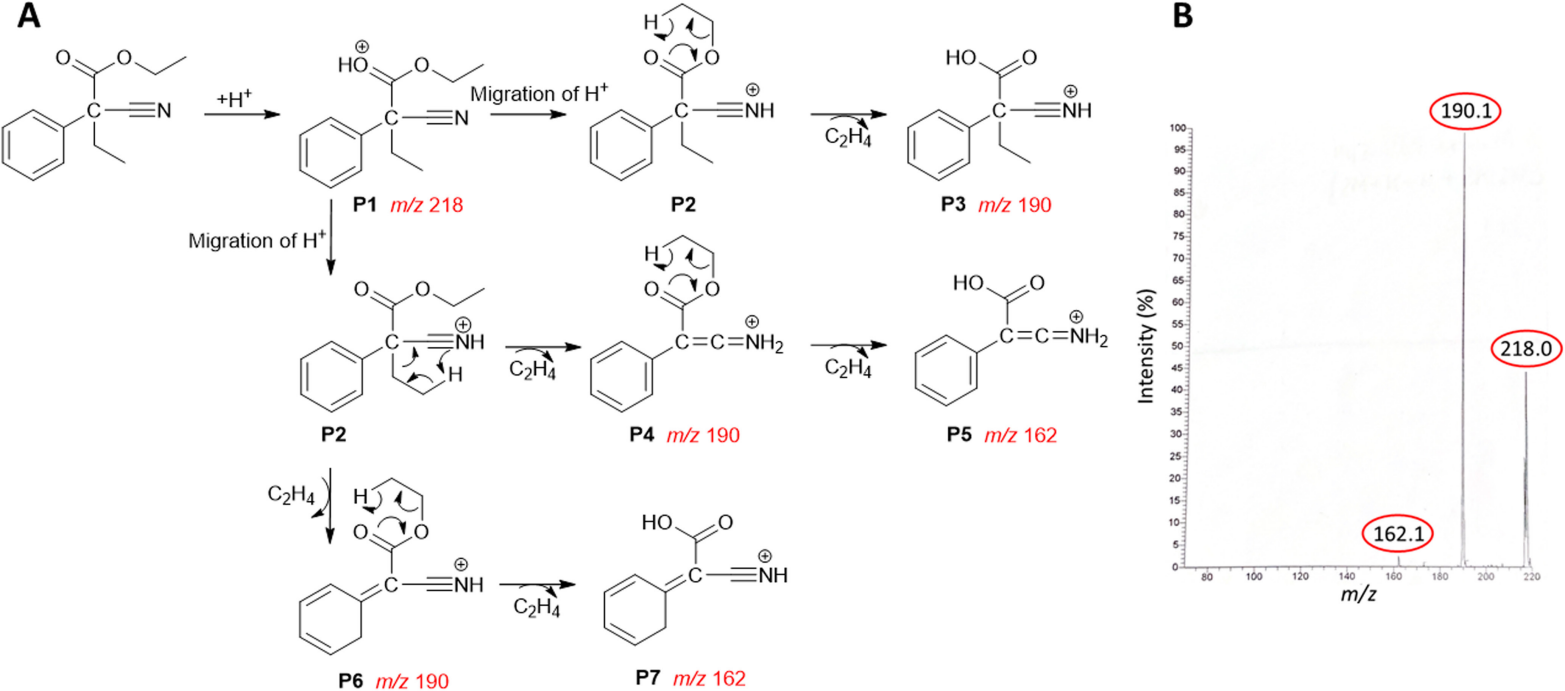
(A) Fragmentation pathway of protonated **4** [M+ H]^+^ predicted by ChemFrag. (B) ESI(+)‐MS^2^ spectrum of **4**. The precursor ion [M+H]^+^ at *m/z* 218 and the marked fragment ions were predicted by ChemFrag.

In the first step, the number of annotated (fragment) ions of 13 compounds shown in Figure S3 and Schemes S2 and S3 was determined using ChemFrag , MetFrag , and CFM‐ID , again considering the absolute number of annotated peaks and the peak intensities ( Table 4 ). ChemFrag achieves a significantly higher annotation rate for these compounds compared with CFM‐ID. A comparison of the total absolute score shows that ChemFrag annotates 58 and MetFrag 49 of the total 79 (fragment) ions for these compounds. In addition, differences can be recognized in the weighted scores. ChemFrag has a total weighted score of 2506/3185, whereas MetFrag has 1991/3185. This difference is probably also related to the missing annotation of the [M+H] ^+^ signals when using MetFrag. To evaluate the data, the fragmentation pathways predicted by ChemFrag were considered. For instance, the predicted reaction pathway of the nitrogen‐containing heterocyclic compound **5** (Scheme S2 ) was compared with the reaction pathway published by Hau et al. [ 25 ] , and it was found that the fragment ions largely coincide. As a further example, the predicted reaction pathway of **4**, a butyric acid derivative, is shown in Scheme 4 (fragmentation path has not yet been published; see Table 3 for ESI‐MS ^2^ data of **4**). Starting from the protonated molecular ion of **4** (**P1**, [C_13_ H_16_ NO_2_ ] ^+^ , *m/z* 218.12), ion **P2** is formed by a proton shift to the ‐C ≡ N group. In the next step, cleavage of the Alk–O bond leads to the formation of the acid **P3** at *m/z* 190.09 ([C_11_ H_12_ NO_2_ ] ^+^ ) and the corresponding alkene C_2_ H_4_ . Starting from **P2**, an alkene elimination from the acid side of the ester via a McLafferty rearrangement involving the ‐C ≡ N group also generates a fragment ion at *m/z* 190.09 (**P4**, [C_11_ H_12_ NO_2_ ] ^+^ ). A subsequent H rearrangement on the alcohol side of the ester and the elimination of the alkene C_2_ H_4_ leads to the acid **P5** ([C_9_ H_8_ NO_2_ ] ^+^ , *m/z* 162.06). A McLafferty rearrangement involving the benzene ring with C_2_ H_4_ cleavage leads to the formation of the fragment ion **P6** at *m/z* 190.09 ([C_11_ H_12_ NO_2_ ] ^+^ ). A further C_2_ H_4_ cleavage leads to the fragment ion **P7** at *m/z* 162.06 ([C_9_ H_8_ NO_2_ ] ^+^ ).

**TABLE 4:** Comparison of the weighted scores and the absolute scores (in brackets) of selected carboxylic acid derivatives, a hydrazine, a thiophosphoric acid ester, sulfonamides, and nitrogen‐, sulfur‐, and oxygen‐containing heterocyclic compounds. The annotation of the (fragment) ions was performed with ChemFrag , MetFrag , and CFM‐ID (scores in the format “determined score/maximum score”; peaks with an rel. intensity ≥ 10% [compounds **4** and **6**] or 5% (all other compounds) are taken into account in the evaluation).

Substance	ChemFrag	MetFrag	CFM‐ID
*N*‐Ethylnicotinamide	165/165 (4/4)	113/165 (3/4)	50/165 (1/4)
2‐Cyano‐2‐phenylbutanoic acid ethyl ester (**4**)	153/157 (3/3)	105/157 (2/3)	47/157 (1/3)
2‐Cyano‐3‐methylhexanoic acid ethyl ester	194/214 (4/5)	30/214 (2/5)	83/214 (1/5)
Nicotinamide (**5**)	195/195 (5/5)	155/195 (3/5)	40/195 (1/5)
2,4‐Diamino‐6‐(hydroxymethyl)pteridine	192/216 (3/4)	185/216 (3/4)	81/216 (1/4)
Gluconic phenylhydrazide	253/260 (8/9)	233/260 (8/9)	23/260 (1/9)
Moxonidine	136/152 (3/5)	121/152 (4/5)	131/152 (2/5)
Hippuric acid methyl ester	203/203 (4/4)	119/203 (3/4)	90/203 (2/4)
*p* ‐Toluenesulfonamide	174/346 (4/7)	180/346 (4/7)	180/346 (4/7)
Sulfamethazine	227/291 (5/8)	103/291 (4/8)	203/291 (5/8)
Thiamethoxam	134/281 (4/9)	202/281 (4/9)	91/281 (3/9)
Quercetin (**6**)	395/395 (7/7)	310/395 (6/7)	45/395 (1/7)
Chlorpyrifos	85/310 (4/9)	135/310 (3/9)	169/310 (4/9)
Total score	2506/3185 (58/79)	1991/3185 (49/79)	1233/3185 (27/79)

In addition, the fragmentation pathway of the flavonoid **6** was predicted as an example of an oxygen*‐*containing heterocyclic compound (Scheme S3 ). The predicted reaction pathway is largely consistent with the studies by Tsimogiannis et al. or Burgert [ 34, 35 ] .

## 4 Conclusion

ChemFrag has been successfully used for the interpretation of ESI(+)‐MS ^n^ fragmentation spectra of antibiotics, pesticides, natural products and structural analogs such as estradiol derivatives. The cleavage and rearrangement rules, implemented in this study, extend the scope of ChemFrag and enable, for example, the annotation of fragment ions of steroids. A comparison with fragmentation pathways published in other studies has shown that ChemFrag provides reliable annotations for compounds such as **1** or **5**. The number of annotated (fragment) ions is comparable or higher than that of the established programs MetFrag and CFM‐ID, whereby fragment ions with higher intensity in particular are annotated. Furthermore, using the example of compound **1**, it was shown that ChemFrag predicts chemically more meaningful annotations than CFM‐ID in some cases. Thus, the combined approach of ChemFrag , using quantum chemistry as well as rule‐based fragmentation, proves to be a valuable addition to established programs.

## Supporting information


**Table S1.** Implemented cleavage rules for various functional groups and structures (for further implemented fragmentation rules see 1)
**Table S2.** Implemented rearrangement rules for various functional groups and structures (for further implemented fragmentation rules see 1)
**Figure S2.** Structures of the molecules shown in Table 1 (see main manuscript)
**Figure S2.** ESI(+)‐MS2 spectrum of estriol 3‐methyl ether (**2**). The precursor ion [M+H]+ at *m/z* 303 and the marked fragment ions were predicted by ChemFrag; *m/z* values used for the calculation of the weighted scores and the absolute scores (see Table 1, main manuscript): *m/z* 303 (100 %), 285 (38 %), 274 (8 %), 267 (70%), 257 (10 %), 241 (16 %), 227 (13 %), 211 (12 %), 199 (10 %), 185 (26 %), 173 (6 %), 171 (11 %), 151 (12 %), 147 (19 %), 135 (14 %), 121 (25 %)
**Figure S3.** Structures of the molecules shown in Table 4 (see main manuscript)
**Scheme S2.** Fragmentation pathway of protonated nicotinamide (**5**) [M+H]+ predicted by ChemFrag [ESI(+)‐ HRMS2 spectrum: see Hau et al.2; detected ions: *m/z* 123 (15 %), 106 (5 %), 80 (100 %), 78 (50 %), 53 (25 %); ions were also used to calculate the weighted scores and the absolute scores (see Table 4, main manuscript)]
**Scheme S3.** Fragmentation pathway of protonated quercetin (**6**) [M+H]+ predicted by ChemFrag [ESI(+)‐MS2 spectrum: see Fig. S4]
**Figure S4.** ESI(+)‐MS2 spectrum of quercetin (**6**). The precursor ion [M+H]+ at *m/z* 303 and the marked fragment ions were predicted by ChemFrag *m/z* values used for the calculation of the weighted scores and the absolute scores: *m/z* 303, 257, 229, 201, 165, 153, 137 (see Table 4, main manuscript)

## Data Availability

The data that support the findings of this study are available from the corresponding author upon reasonable request.
