# Supplementary material for: Prediction of Fragmentation Pathway of Natural Products, Antibiotics, and Pesticides by ChemFrag
Source: J Mass Spectrom. 2025 Apr 7;60(5):e5129. doi: 10.1002/jms.5129 (PMC11976197; doi:10.1002/jms.5129)
Supplement: Supplementary file 1 — Table S1. Implemented cleavage rules for various functional groups and structures (for further implemented fragmentation rules see 1) Table S2. Implemented rearrangement rules for various functional groups and structures (for further implemented fragmentation rules see 1) Figure S2. Structures of the molecules shown in Table 1 (see main manuscript) Figure S2. ESI(+)‐MS2 spectrum of estriol 3‐methyl ether (2). The precursor ion [M+H]+ at m/z 303 and the marked fragment ions were predicted by ChemFrag; m/z values used for the calculation of the weighted scores and the absolute scores (see Table 1, main manuscript): m/z 303 (100 %), 285 (38 %), 274 (8 %), 267 (70%), 257 (10 %), 241 (16 %), 227 (13 %), 211 (12 %), 199 (10 %), 185 (26 %), 173 (6 %), 171 (11 %), 151 (12 %), 147 (19 %), 135 (14 %), 121 (25 %) Figure S3. Structures of the molecules shown in Table 4 (see main manuscript) Scheme S2. Fragmentation pathway of protonated nicotinamide (5) [M+H]+ predicted by ChemFrag [ESI(+)‐ HRMS2 spectrum: see Hau et al.2; detected ions: m/z 123 (15 %), 106 (5 %), 80 (100 %), 78 (50 %), 53 (25 %); ions were also used to calculate the weighted scores and the absolute scores (see Table 4, main manuscript)] Scheme S3. Fragmentation pathway of protonated quercetin (6) [M+H]+ predicted by ChemFrag [ESI(+)‐MS2 spectrum: see Fig. S4] Figure S4. ESI(+)‐MS2 spectrum of quercetin (6). The precursor ion [M+H]+ at m/z 303 and the marked fragment ions were predicted by ChemFrag m/z values used for the calculation of the weighted scores and the absolute scores: m/z 303, 257, 229, 201, 165, 153, 137 (see Table 4, main manuscript) [file JMS-60-e5129-s001.pdf]

Supplementary Material

Prediction of Fragmentation Pathway of Natural Products, Antibiotics and Pesticides by ChemFrag

Jördis-Ann Schüler[*1], Annemarie E. Kramell[2], Antonia Schmidt[1], Pauline D. Walesch[1], and René Csuk[2]

[1]Institute of Computer Science, Martin Luther University Halle-Wittenberg, Von-Seckendorff-Platz 1, 06120 Halle (Saale), Germany

[2]Department of Organic Chemistry, Martin Luther University Halle-Wittenberg, Kurt-Mothes-Str. 2, 06120 Halle (Saale), Germany

***Corresponding author**: Dr. Jördis-Ann Schüler, Institute of Computer Science, Martin Luther University Halle-Wittenberg, 06120 Halle (Saale), Germany

E-mail: joerdis-ann.schueler@informatik.uni-halle.de

Table S1. Implemented cleavage rules for various functional groups and structures (for further implemented fragmentation rules see [1])

ID	Rule	SMARTS	Steps/remarks	Scheme
1	Ammoniak removal/ formation of an acyl ion - Amides	[C,c,O,N](=O)[NH3+] or C(=O)[N,nH,N+][CH2+,C,CH3]	- Bond between C and O atom becomes a triple bond<br>- Charge is located on the O atom<br>- R^2 and R^3 can be H atoms or alkyl groups	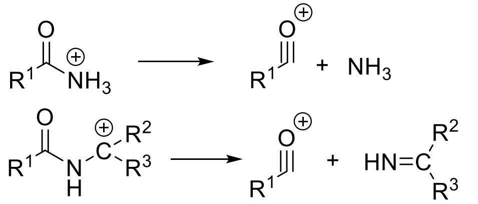
2	Elimination of the carbamoyl group - Nicotinamide	[nH+][c][c][C](=O)N	The Elimination is shown using the example of nicotinamide	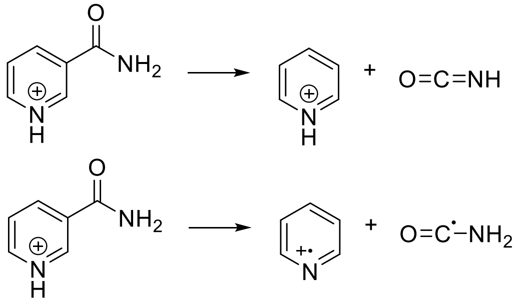
3	Ammoniak/ amines and SO_2 removal – Sulfonamides, Sulfonyl group	S(=O)(=O)[N+] and [C,c,N,n,S,s,O,o][S+](=O)=O	- Three H atoms are bound to the N atom, but organic residues can also be bound instead of the H atoms<br>- Prerequisite: R is not charged before the loss of SO_2<br>- R = C, N, S, O; R can be aliphatic or aromatic	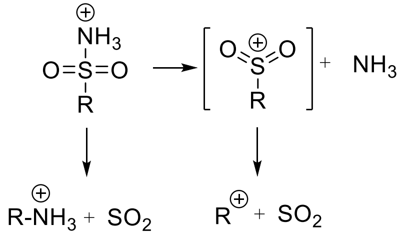
4	Formation of quaternary ammonium cations - Sulfonamides	[N]c1ccc([S](=O)(=O)[N+])cc1	R^1, R^2 and R^3 can be H atoms or alkyl groups	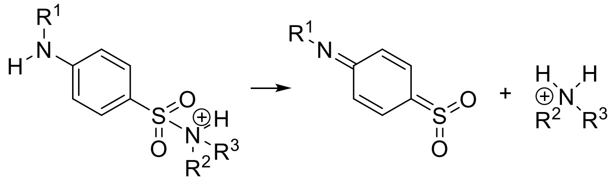
5	Elimination of H_2O - - Sulfonamides	[S+](=[O])([OH])[N;!H0]	Loss of H_2O occurs after tautomerization of the molecular ion	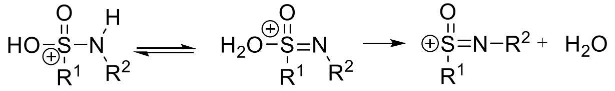

Table S1. Continued

ID	Rule	SMARTS	Steps/remarks	Scheme
6	Elimination of SO - Sulfonyl group	[C][S+](=[O])=[N,O]	X = O, N	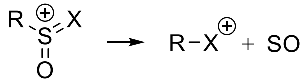
7	Elimination of SO - Formation of quinonimines	[N]c1ccc([S+](=O)=O)cc1		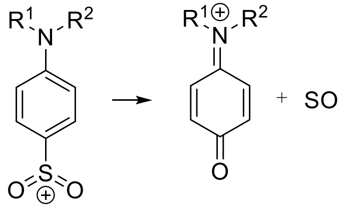
8	Aromatic elimination	C=CC=CC=CC=C	- R^2 is usually the positively charge site<br>- The aromatic ring can be substituted and thus also carry a charge	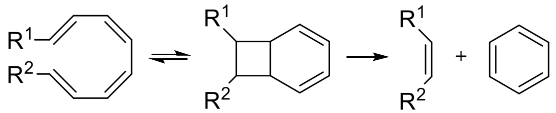
9	Elimination of acetylene - Benzene	[c][c]	- Both C atoms are part of a benzene ring and a H atom is bound to the C atoms	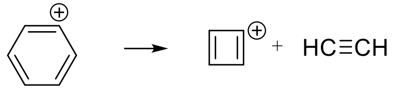
10	Benzyl cleavage	[Br,C,Cl,N,O]C[C]1[C]=[C][C]=[C][C]1	- Prerequisite: either a_1 or a_6 is charged, and either a_1 or a_6 has at least one free electron<br>- X = Br, Cl, C, N, O<br>- the loss of $Br^•$ or $Cl^•$ occurs preferentially with bromo or chloro compounds	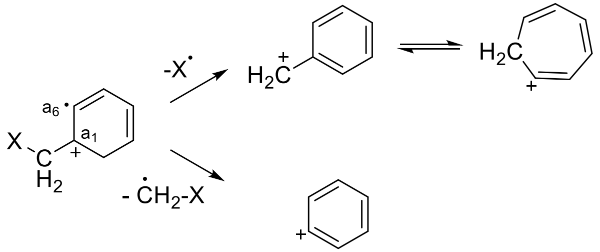
11	Elimination of acid HX (X: F, Cl, Br, I) – Halogen compounds	[F, Cl,Br,I]	- Iteration for atoms bound to the halogen atom:<br>1: R gets single electron (homolytic cleavage) – loss of halogen radical (the loss of $Br^•$ is more common in bromo compounds)<br>2: halogen atom gets bonding electron pair (heterolytic cleavage) – loss of HX (neutral loss) and formation of R^+	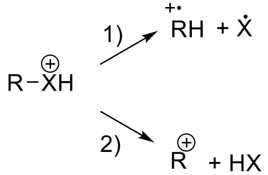

Table S1. Continued

ID	Rule	SMARTS	Steps/remarks	Scheme
12	CO and H_2O removal - Carboxylic acids	[CH2][C]([OH])(=[OH+])	Aliphatic carboxylic acids: CO + H_2O elimination	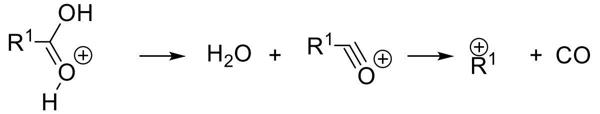
13	Fragmentation of esters	[C,c][O+][C,c]([C,c])=O	- 1 and 2: Depending on the proton affinity of the products it leads to the protonated acid $[R^1COOH + H]^+$ or to the alkyl cation $[R^2]^+$<br>- 3 and 4: α-Cleavage with subsequent CO elimination<br>- alkene elimination via McLafferty rearrangements on both sides of the carboxyl group as a further possibility	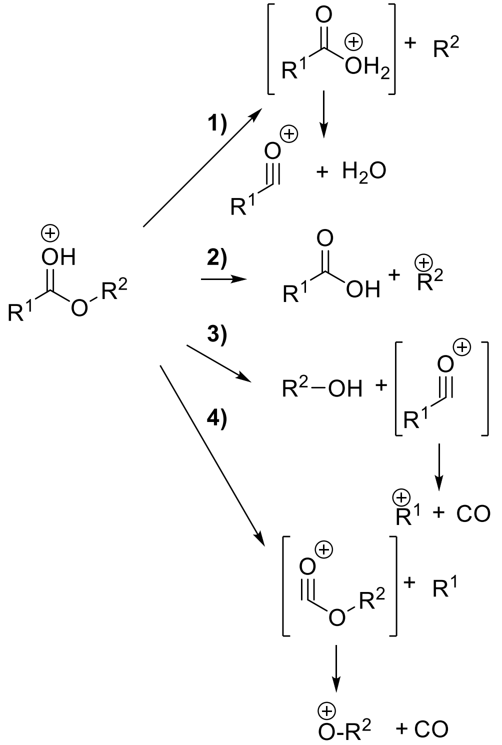

Table S1. Continued

ID	Rule	SMARTS	Steps/remarks	Scheme
14	CO removal - Ketones	[C,c]=[O,o]; [C,c]#[O,o]	- Prerequisites: (a) presence of a cyclic, highly unsaturated ketone; (b) presence of an acyl ion (bond next to CO was cleaved)<br>- Neutral loss (CO) in both cases	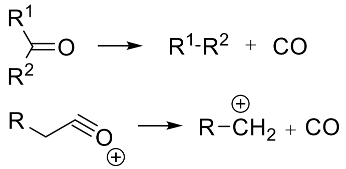
15	Elimination of CO - Quinones	c1ccc2c(c1)C(=O)c3cccc c3C2=O	e.g. anthraquinone: loss of 1 or 2 equivalents of CO	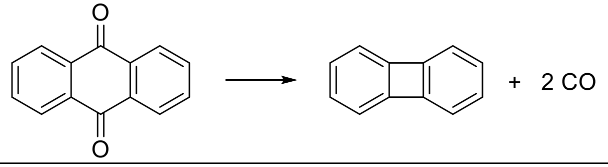
16	Elimination of HNC (hydrogen isocyanide)	[N]=C1[C][C]=[C][C]=[C]1; [N]c1[c][c][c][c][c]1	- Elimination of HNC from aniline<br>- Loss of methyl radical by homolytic cleavage of the C–N bond in methyl amines	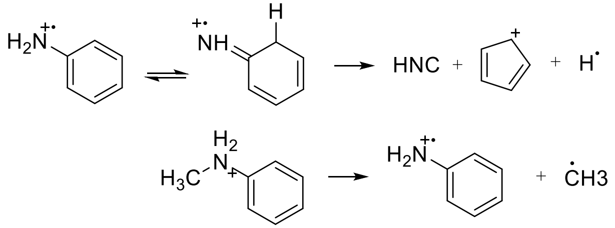

Table S1. Continued

ID	Rule	SMARTS	Steps/remarks	Scheme
17	CO removal – Phenols and alkyl/ aryl ethers	[N,C]=[C](O)-[C]=[C]; [n,c][c](O)[c][c]	- Loss of CO occurs after tautomerization of the molecular ion of the phenolic compound<br>- Loss of CO, H_2O, alkenes or CHO· can also be observed with correspondingly substituted heteroaromats	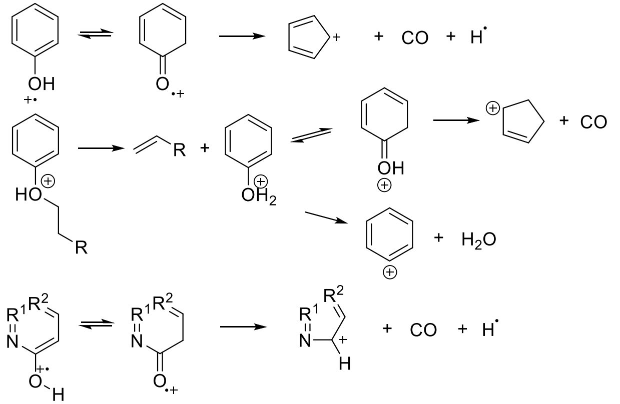
18	Ethen removal – Ethoxy group	O-[CH2]-[CH3]	- Prerequisite: O atom is bound to 2 C atoms;<br>- Cleavage of the C–O bond, results either in a carbocation or, by ethen elimination, in a protonated alcohol	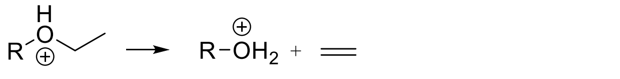
19	Elimination of R-OH - Ethers	[OH+]	Depending on the proton affinities of the products, it results either in a carbocation or, by alkene elimination, in a protonated alcohol $[R^1+OH + H]^+$/ $[R^2+OH + H]^+$	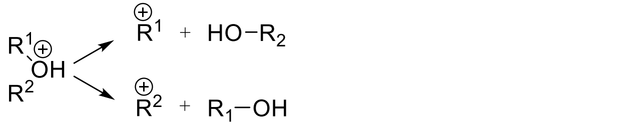
20	Inductive cleavage	C[O+,N+]C; C[O+,N+]	Charge-induced cleavage from a radical cation and a cation;<br>Y = O, N; R^1 and R^2: alkyl groups	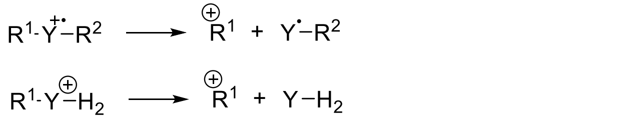

Table S1. Continued

ID	Rule	SMARTS	Steps/remarks	Scheme
21	α-Cleavage – Amines and ethers	[C][N,O][C]; [C]C([C])=[N,O]; [C][C]([C])[N]; [C][C]O	- Cleavage of the C-C bond next to the N or O atom, larger substituents are eliminated preferably;<br>- X = N, O (one or two further subsituents are bound to the N/O atom)	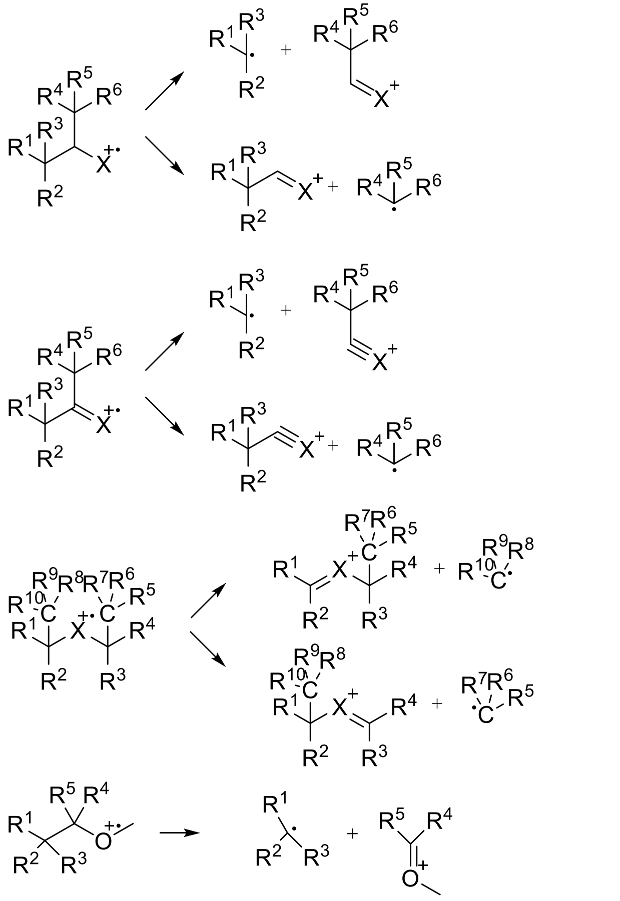

Table S1. Continued

ID	Rule	SMARTS	Steps/remarks	Scheme
22	Onium reaction	C=[N+,O+,S+]CC	- Prerequisite: catioinic fragment ion with a heteroatom that carries the charge<br>- Cleavage of an alkyl group (at least one C_2 chain) from the hetero atom of the ion with transfer of a H atom from this group to the hetero atom	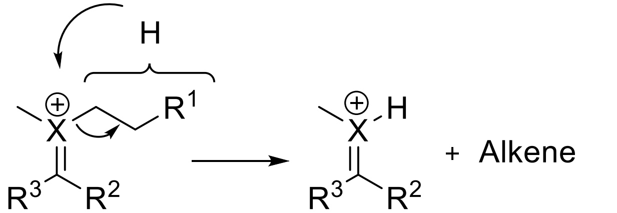
23	retro-Diels–Alder reaction (RDA) – Cyclohexenes/ bicyclic hydrocarbons	[C,N,O]1=[C,N,O][C,N,O][C,N,O][C,N,O][C,N,O]1;<br>[c,n,o]12[c,n,o]([c,n,o][c,n,o][c,n,o][c,n,o]2)[C,N,O][C,N,O][C,N,O][C,N,O]1	- Prerequisite: presence of a cation/ the compound is not neutral<br>- An ion and a neutral compound are formed<br>- RDA reaction of bicyclic hydrocarbons is shown using the examples of tetrahydroisoquinoline and 1-tetralone	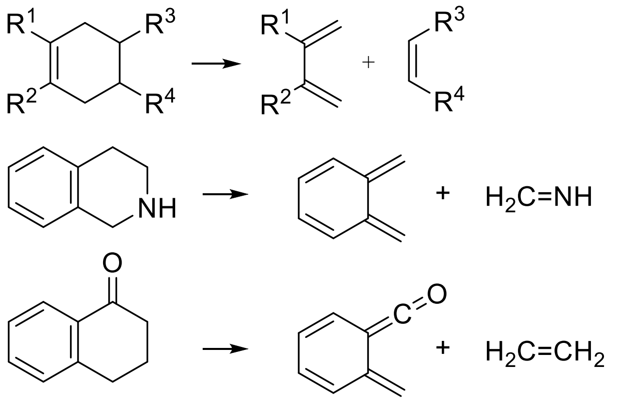

Table S1. Continued

ID	Rule	SMARTS	Steps/remarks	Scheme
24	McLafferty rearrangement	[C,N,O,c]=[C,N,O,c][C,N,O][C,N,O][C,N,O];[C,N,O][C,N,O][C,N,O][C,N,O]=[C,N,O]	- Both radical and non-radical cations can be used as starting ions<br>- Also occurs with aromatic compounds (1,5-H$^{\bullet}$ shift to the aromatic system + β-cleavage)	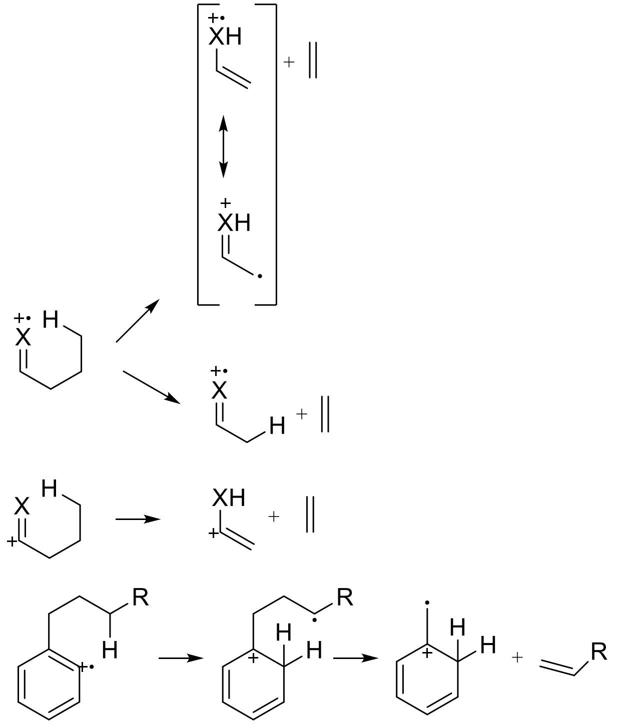
25	McLafferty rearrangement – aliphatic nitriles	[NH+]#CCC[CH]	- McLafferty rearrangement yielding $CR_2{=}C{=}NH_2^+$<br>- R^1, R^2 and R^3 can be H atoms or alkyl groups	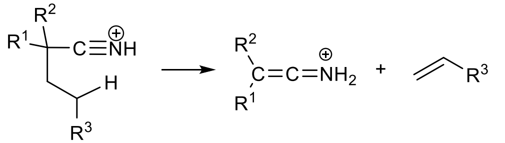
26	retro-En reaction -	[C,O,N,S]=[C][C][C][C;!H0]	X = C, O, N, S	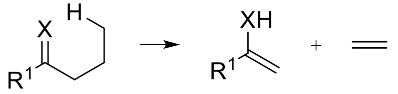

Table S1. Continued

ID	Rule	SMARTS	Steps/remarks	Scheme
27	Alkane/alkene elimination – Branched alkyl groups	[C,O,N][C,O,N][C,O,N]	- Prerequisite: C1/A atom binds at least one H atom<br>- A,B,X can also be N or O atoms	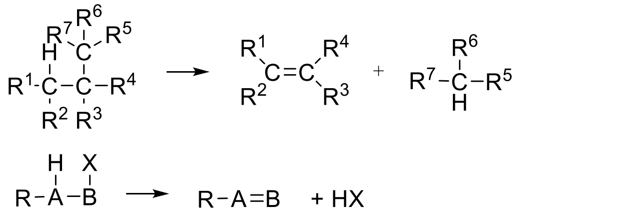
28	Elimination of CHCl - Moxonidine	[N,n][C,c](Cl)[C,c]	The Elimination is shown using a fragment ion of moxonidine	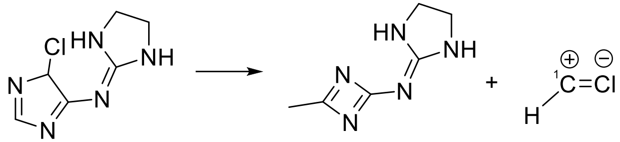
29	Elimination of a hydrogen radical	[C][N+,O+]	- X = N, O<br>- Alternatively, the α-cleavage can also be carried out by eliminating an alkyl radical	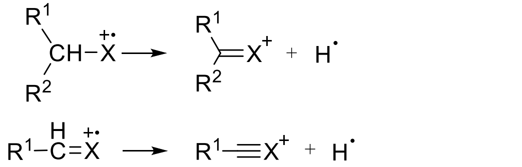
30	Cleavage of steroids	[C,c]1CCC2C([C,c]1)CC[C+]3CCCC23	Prerequisites: at least one H atom is bound to C4, C7 and C5; one H atom is removed from these C atoms	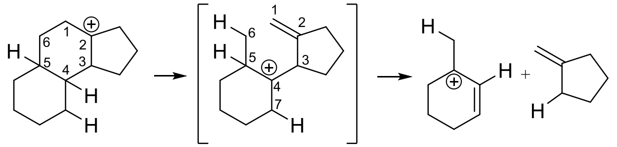
31	Elimination of HCN -	[NH+]2(=CC=CC=C2)		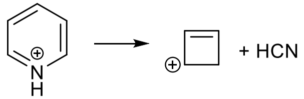

Table S2. Implemented rearrangement rules for various functional groups and structures (for further implemented fragmentation rules see [1])

ID	Rule	SMARTS	Steps/remarks	Scheme
1	Resonance structures – Ions with positively and negatively charged groups	[O-][C,C+,N,N+,S,S+]=[C,N,S,O]; [N-]=[C]=[C,N,S,O] or O=[C,C+,N,N+,S,S+][C-,N-,S-,O-]; N#[C][C-,N-,S-,O-]	R^1, R^2 or R^3 is positively charged	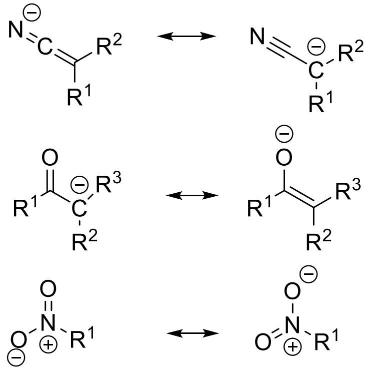
2	Formation of double and triple bonds - Carbenes	[C,c][C,c]	Prerequisites: (a) the atom C1 carries a lone pair; at least one H atom is bound to the C2 atom (or vice versa) (b) the atoms C1 and C2 each have a lone pair	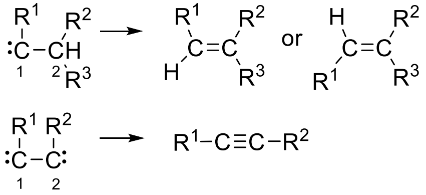
3	Formation of double and triple bonds - N-containing compounds	[C,c][N,n] or [C+][N]	Prerequisites: (a) the atoms N and C each have a lone pair (b) N and C both carry a single electron (c) N atom carries a negative charge	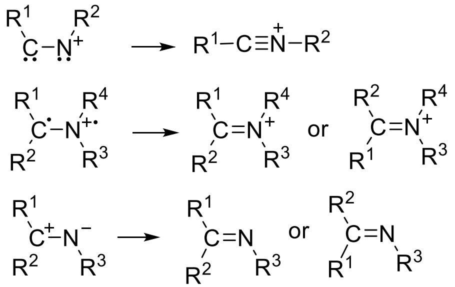

Table S2. Continued

ID	Rule	SMARTS	Steps/remarks	Scheme
4	Tautomerization - Phenol	[O]c1[c][c][c][c][c]1	Keto-enol tautomerization	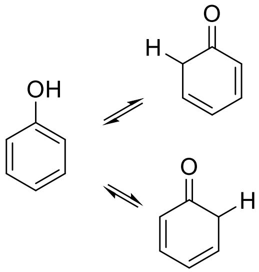
5	Formation of a carbonyl group	[C,c][O]	Prerequisites: the C atom carries a a lone pair and at least one H atom binds to the O atom	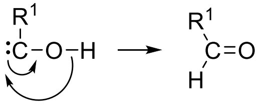
6	Rearrangement of an acyl ion	[C+]=[O]		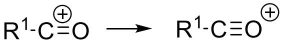
7	Cyclization - Formation of cyclic ethers and amines	[C][C]c1[c][c][c][c]c1[C]; [C]c1[c][c][c][c]c1[C][O,N]	Prerequisites: R must be involved in a double or triple bond and carry a positive charge (see example); the X atom carries a a lone pair;<br>X = C, N, O	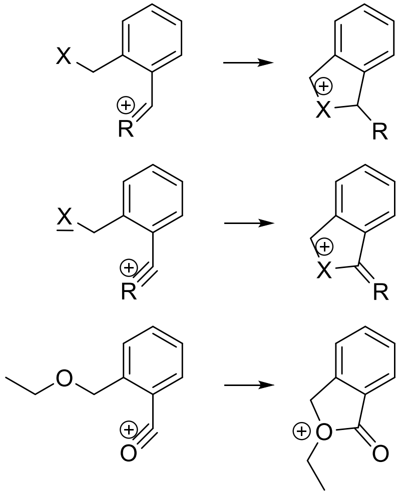

Table S2. Continued

ID	Rule	SMARTS	Steps/remarks	Scheme
8	Cyclization - Formation of cyclic thioethers	[O+,OH+]=[C,c,N,n,S,s][C,c,N,n,O,o,S,s][C,c,N,n,O,o,S,s][C,c,N,n,O,o,S,s][S]	Alkyl groups can also be bound to the O and S atoms instead of the H atoms	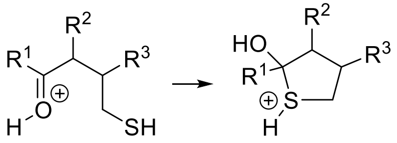
9	Shift of H$^{\bullet}$	[C][N+,O+] or [N+][C,N,O][CH3]	Prerequisites: (a) the X atom carries one unpaired valence electron and a positive charge; at least one H atom binds to the C atom, X = N, O; (b) the N atom carries one unpaired valence electron and a positive charge	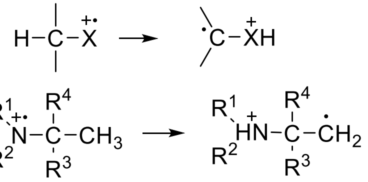
10	Methyl shift	[CH3]C12CCCC1CC[C+]2; [CH3]C12CCCCC1CC[C+]2	see main manuscript	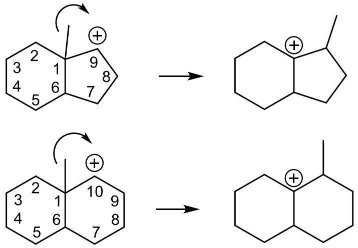
11	Pericyclic shift – [1,3] and [1,5] Shift	[C][C]=[C][C]=[C]	The migration of a σ-bond adjacent to one or more π systems to a new position	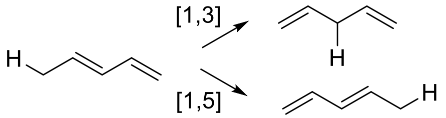

Table S2. Continued

ID	Rule	SMARTS	Steps/remarks	Scheme
12	H^+ shift – Iminium ions and carbocations	[N+,n+]=[C,c]-[C,c] or [C+,c+][C,c]	- Intermolecular and intramolecular proton shift<br>- Migration of H^+ and formation of an alkene, alkyne, iminium ion (X = N, O)	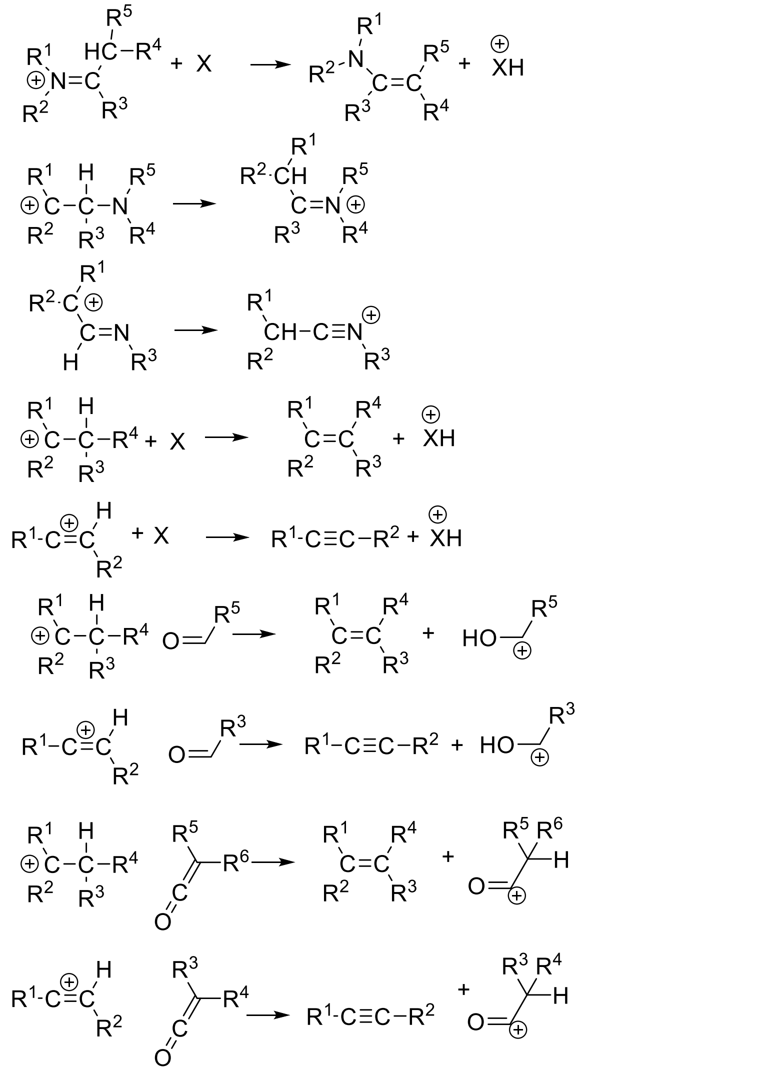

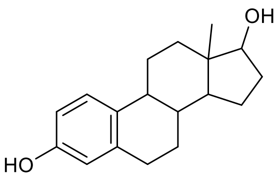

17*β*-Estradiol (**1**)

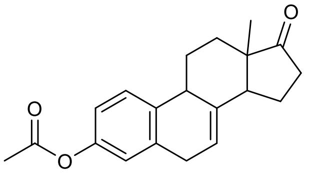

Equilin 3-acetate

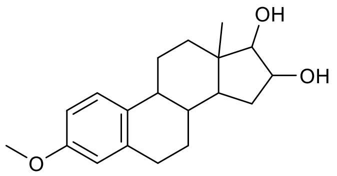

Estriol 3-methyl ether (**2**)

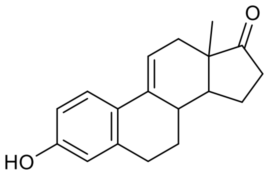

9(11)-Dehydroestrone

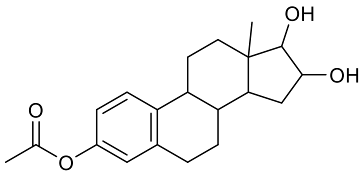

Estriol 3-acetate

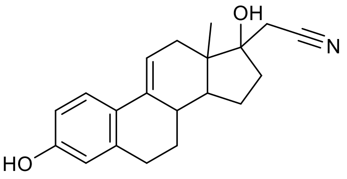

Δ[9,11]-Dehydro-17*α*-cyanomethyl-estradiol (**3**)

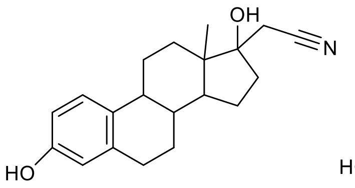

17*α*-Cyanomethyl-estradiol

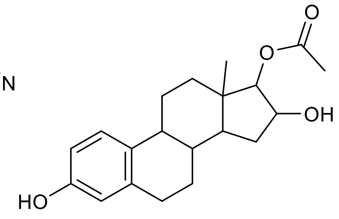

Estriol 17-acetate

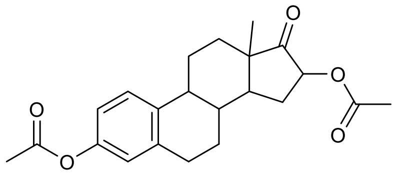

α-Hydroxyestrone diacetate

Figure S2. Structures of the molecules shown in Table 1 (see main manuscript)

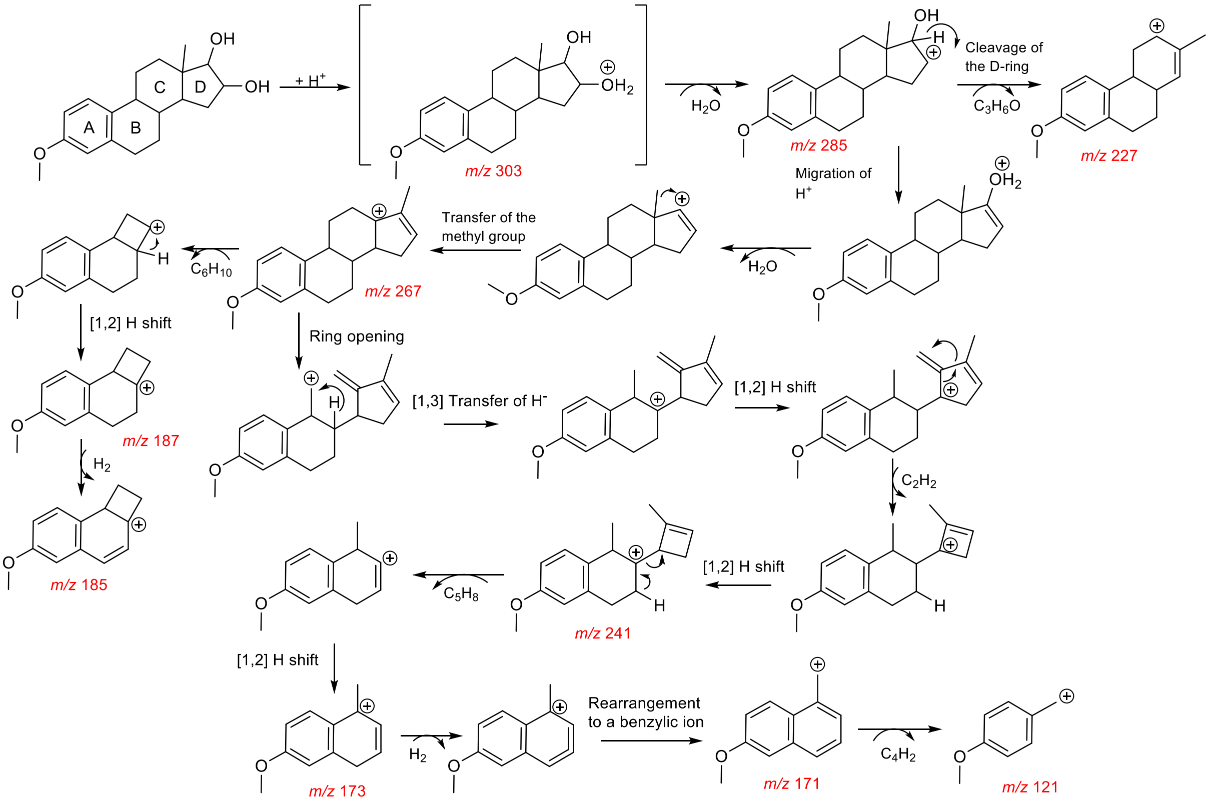

Scheme S1. Fragmentation pathway of protonated estriol 3-methyl ether (**2**) $[M+H]^+$ predicted by `ChemFrag` (ESI(+)-MS^2 spectrum: see Fig. S2)

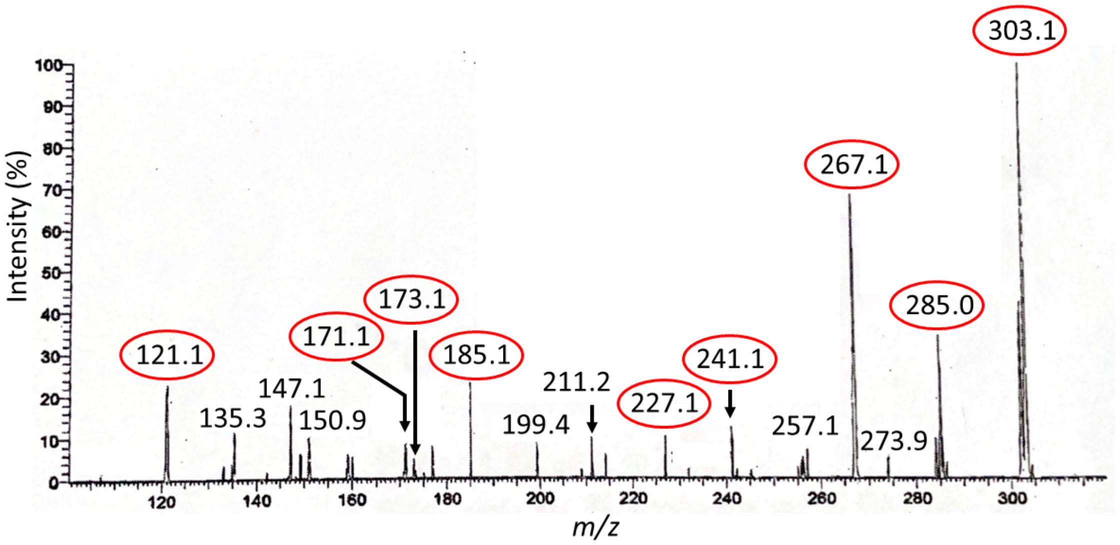

Figure S2. ESI(+)-MS^2 spectrum of estriol 3-methyl ether (**2**). The precursor ion $[M+H]^+$ at *m/z* 303 and the marked fragment ions were predicted by ChemFrag; *m/z* values used for the calculation of the weighted scores and the absolute scores (see Table 1, main manuscript): *m/z* 303 (100 %), 285 (38 %), 274 (8 %), 267 (70%), 257 (10 %), 241 (16 %), 227 (13 %), 211 (12 %), 199 (10 %), 185 (26 %), 173 (6 %), 171 (11 %), 151 (12 %), 147 (19 %), 135 (14 %), 121 (25 %)

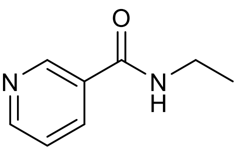

N-Ethylnicotinamide

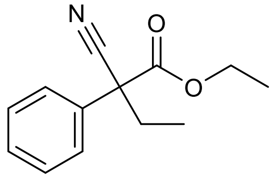

2-Cyano-2-phenylbutanoic acid ethyl ester (**4**)

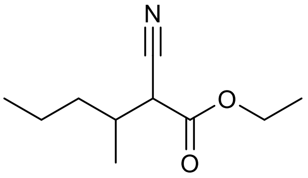

2-Cyano-3-methylhexanoic acid ethyl ester

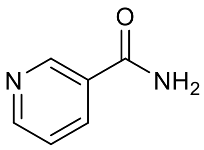

Nicotinamide (**5**)

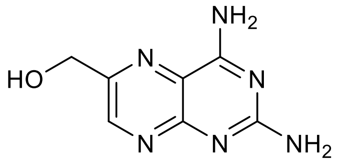

2,4-Diamino-6-(hydroxylmethyl)pteridine

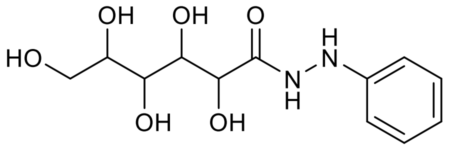

Gluconic phenylhydrazide

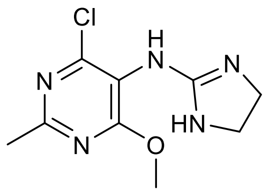

Moxonidine

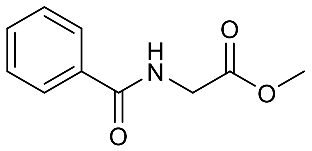

Hippuric acid methyl ester

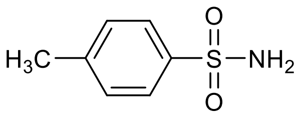

p-Toluenesulfonamide

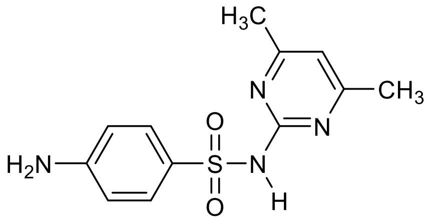

Sulfamethazine

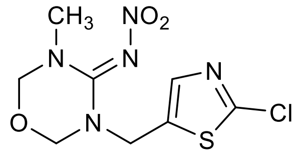

Thiamethoxam

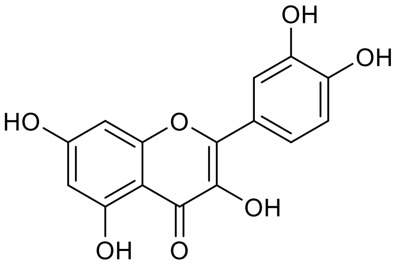

Quercetin (**6**)

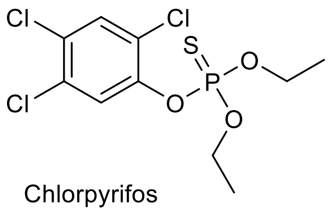

Chlorpyrifos

Figure S3. Structures of the molecules shown in Table 4 (see main manuscript)

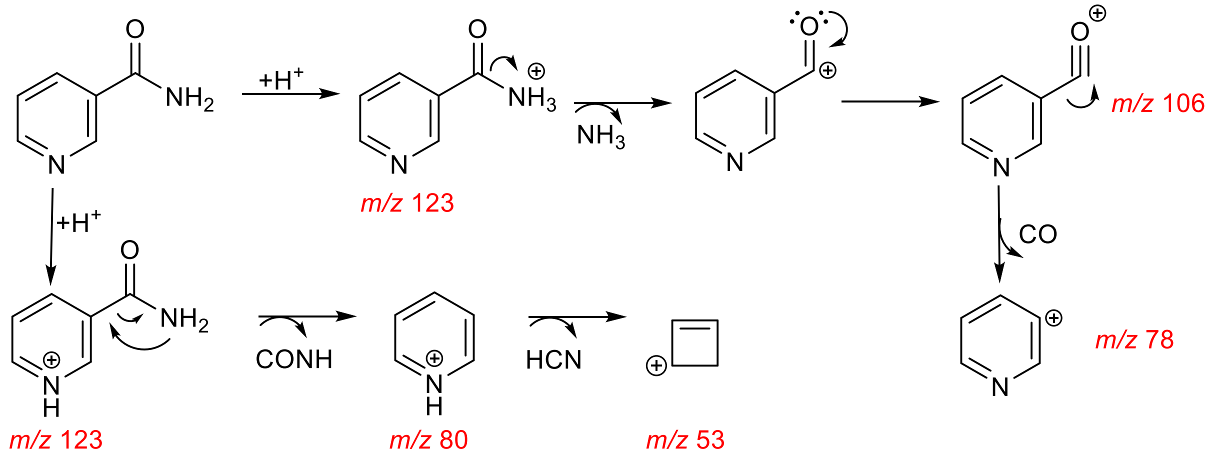

Scheme S2. Fragmentation pathway of protonated nicotinamide (**5**) $[M+H]^+$ predicted by `ChemFrag` [ESI(+)-HRMS^2 spectrum: see Hau *et al.*[2]; detected ions: *m/z* 123 (15 %), 106 (5 %), 80 (100 %), 78 (50 %), 53 (25 %); ions were also used to calculate the weighted scores and the absolute scores (see Table 4, main manuscript)]

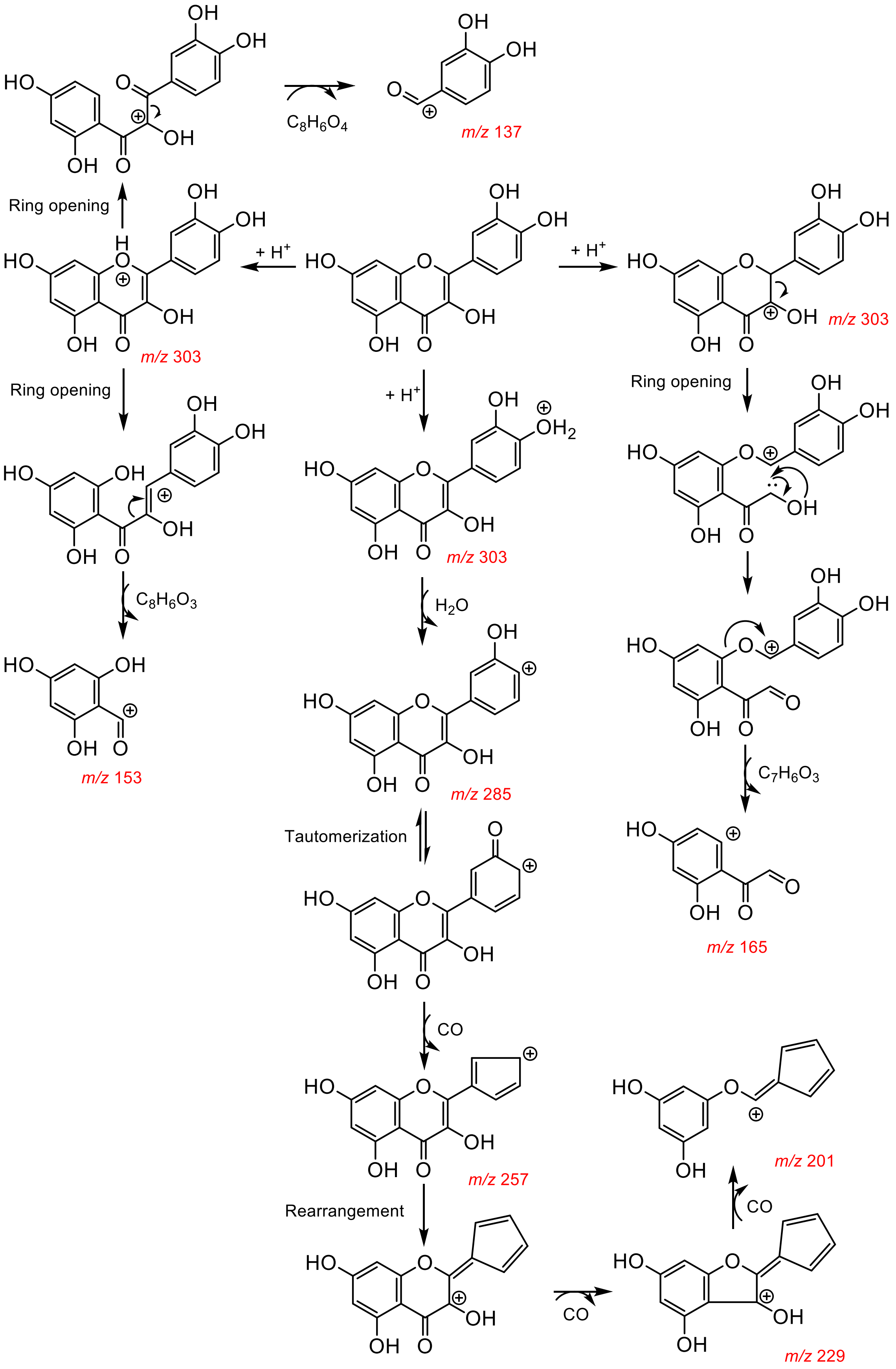

Scheme S3. Fragmentation pathway of protonated quercetin (**6**) $[M+H]^+$ predicted by `ChemFrag` [ESI(+)-MS^2 spectrum: see Fig. S4]

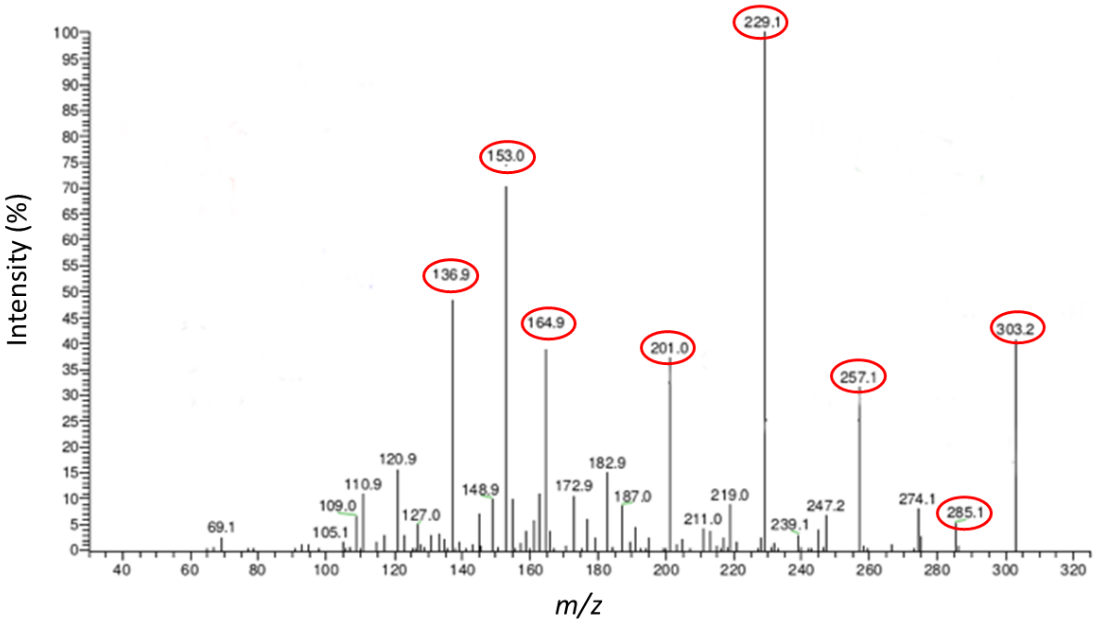

Figure S4. ESI(+)-MS^2 spectrum of quercetin (**6**). The precursor ion $[M+H]^+$ at *m/z* 303 and the marked fragment ions were predicted by ChemFrag *m/z* values used for the calculation of the weighted scores and the absolute scores: *m/z* 303, 257, 229, 201, 165, 153, 137 (see Table 4, main manuscript)

References

1. Schüler J.-A., Neumann S., Müller-Hannemann M., Brandt W. ChemFrag: Chemically meaningful annotation of fragment ion mass spectra. *J Mass Spectrom.* **2018**, *53*(11), 1104-1115. doi:10.1002/jms.4278
2. Hau J., Stadler R., Jenny T.A., Fay L.B. Tandem mass spectrometric accurate mass performance of time-of-flight and Fourier transform ion cyclotron resonance mass spectrometry: a case study with pyridine derivatives. *Rapid Commun Mass Spectrom.* **2001**, *15*(19), 1840-1848. doi:10.1002/rcm.444
